# The Plant-Derived* Bauhinia bauhinioides* Kallikrein Proteinase Inhibitor (rBbKI) Attenuates Elastase-Induced Emphysema in Mice

**DOI:** 10.1155/2016/5346574

**Published:** 2016-08-01

**Authors:** Bruno Tadeu Martins-Olivera, Rafael Almeida-Reis, Osmar Aparecido Theodoro-Júnior, Leandro Vilela Oliva, Natalia Neto dos Santos Nunes, Clarice Rosa Olivo, Marlon Vilela de Brito, Carla Máximo Prado, Edna Aparecida Leick, Mílton de Arruda Martins, Maria Luiza Vilela Oliva, Renato Fraga Righetti, Iolanda de Fátima Lopes Calvo Tibério

**Affiliations:** ^1^Department of Clinical Medicine, School of Medicine, University of São Paulo, 01246-903 São Paulo, SP, Brazil; ^2^Department of Biochemistry, Universidade Federal de São Paulo, 04044-020 São Paulo, SP, Brazil; ^3^Department of Bioscience, Federal University of Sao Paulo, 11015-020 Santos, SP, Brazil

## Abstract

*Background*. Elastase mediates important oxidative actions during the development of chronic obstructive pulmonary disease (COPD). However, few resources for the inhibition of elastase have been investigated. Our study evaluated the ability of the recombinant plant derived* Bauhinia bauhinioides* Kallikrein proteinase Inhibitor (rBbKI) to modulate elastase-induced pulmonary inflammation.* Methods*. C57Bl/6 mice were given intratracheal elastase (ELA group) or saline (SAL group) and were treated intraperitoneally with rBbKI (ELA-rBbKI and SAL-rBbKI groups). At day 28, the following analyses were performed: (I) lung mechanics, (II) exhaled nitric oxide (ENO), (III) bronchoalveolar lavage fluid (BALF), and (IV) lung immunohistochemical staining.* Results*. In addition to decreasing mechanical alterations and alveolar septum disruption, rBbKI reduced the number of cells in the BALF and decreased the cellular expression of TNF-*α*, MMP-9, MMP-12, TIMP-1, eNOS, and iNOS in airways and alveolar walls compared with the ELA group. rBbKI decreased the volume proportion of 8-iso-PGF2*α*, collagen, and elastic fibers in the airways and alveolar walls compared with the ELA group. A reduction in the number of MUC-5-positive cells in the airway walls was also observed.* Conclusion*. rBbKI reduced elastase-induced pulmonary inflammation and extracellular matrix remodeling. rBbKI may be a potential pharmacological tool for COPD treatment.

## 1. Introduction

Chronic obstructive pulmonary disease (COPD) is a preventable and treatable disease characterized by chronic airflow limitation that is not fully reversible [1]. COPD patients exhibit an abnormal inflammatory response to particles or toxic gases causing several pathological changes in the lung along with important comorbidities that can contribute to disease severity [2].

The chronic pulmonary inflammatory process in COPD is caused by smoking. It is important to emphasize that the inflammatory response does not cease after smoking cessation [3]. The inflammatory response in COPD results in large and small airways becoming inflamed, goblet cell hyperplasia, and an increase in mucosal glands [4]. The primary cells involved in this process are neutrophils, macrophages, and CD8+ lymphocytes in the upper airways, particularly in advanced COPD [5].

Lung tissue alterations in COPD are the result of an imbalance between proteinases and antiproteinases. There is evidence to support that the inhalation of cigarette smoke induces an increase in neutrophils and macrophages which release enzymes such as cathepsin B, K, L, and S, neutrophil elastase, proteinase 3, and matrix metalloproteinases 9 and 12. These enzymes are not completely inhibited by antiproteinases, leading to the destruction of connective tissue [6, 7].

The COPD treatment approach is divided into the following steps: reduction of risk factors, therapeutic management of stable disease, and therapeutic management of disease exacerbations [8]. Notably, no currently available treatment reduces disease progression or suppresses inflammation in small airways and lung parenchyma. To better study the pathogenesis of emphysema, the elastase-induced animal model was developed 30 years ago. It is a simple and widely accepted emphysema animal model and is used to elucidate the mechanisms involved in emphysema pathophysiology and to examine new pharmacological approaches [4, 9].

Proteinase inhibitors inhibit proteolytic enzymes or increase levels of endogenous antiproteinases and may contribute to the prevention of disease progression [10]. Proteinases are signaling molecules involved in hemostasis, cell death, cell proliferation, DNA replication, the inflammatory response, and tissue remodeling [11]. Protease inhibitors are also present in plants and are involved in plant biological functions and pathological conditions. Consequently, the prevention of undesired proteolysis has been extensively studied [12, 13].

*Bauhinia* is a plant genus from the subfamily Caesalpinioideae which comprises over 600 species native to tropical and subtropical forests [14]. Numerous proteinase inhibitors have been isolated from this genus, particularly from the* Bauhinia bauhinioides* species. In the present study, we evaluated the recombinant plant-derived the* Bauhinia bauhinioides* kallikrein Inhibitor (rBbKI) [15].

Kallikreins are glycoproteins present in glandular cells, neutrophils, and biological fluids. Human tissue kallikreins are glycoproteins expressed in cells and tissues with specific regulatory functions such as ion transport, glucose transport in synergy with insulin, and regulation of blood flow through the formation of kinins [13]. Elevated tissue levels of kinin and kallikrein have been associated with hypertension and diabetes [16].

BbKI, or its recombinant form rBbKI, inhibits trypsin, plasmin, and chymotrypsin. This protein is also a potent inhibitor of human plasma kallikrein (HuPk). Inhibition of HuPK by BbKI/rBbKI causes a decrease in bradykinin release due to the decreased availability of HuPK. BbKI is the first plant-derived inhibitor of the primary structure set described that is an inhibitor of tissue kallikrein [17, 18]. Indeed, rBbKI was effective in inhibiting the viability of tumor cell lines [19].

The present study aimed to investigate whether rBbKI contributes to the inactivation of elastase-induced pulmonary mechanical alteration, inflammatory response, extracellular matrix remodeling, and oxidative stress responses.

## 2. Methods

### 2.1. Animals

Male C57Bl/6 mice (20–25 g) were maintained in an animal facility with a 12-hour light-dark cycle and were offered water and chow* ad libitum*. The animals received humane care in compliance with the “Guide for Care and Use of Laboratory Animals” (National Institutes of Health, publication 86-23, revised 1985), and the study protocol was approved by the Ethical Committee of the University of São Paulo.

### 2.2. Inhibitor Purification

**Figure 1:**
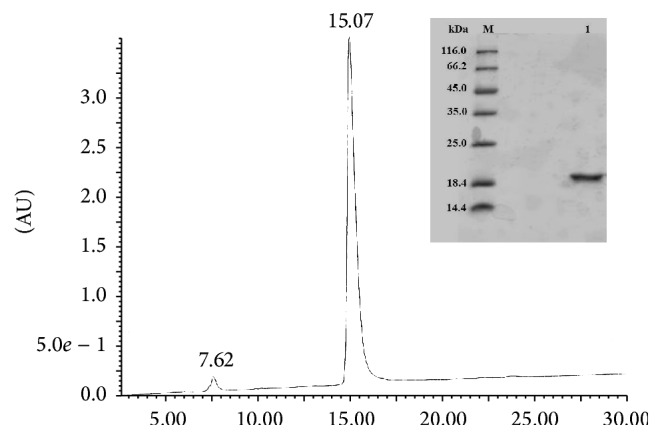
Reverse phase chromatograph, HPLC system *μ*-Bondapak C18. SDS-PAGE (15%) from reverse phase chromatography on C18. (M) Molecular mass standards; (1) rBbKI (30 *µ*g) treated with *β*-mercaptoethanol.

Overexpression of the rBbKI in* E. coli* BL21(DE3) cells and its purification were carried according to Araújo et al. [15] modified by Zhou et al., [20]. Briefly, cells containing the target gene cloned into the expression vector pET28a (Novagen) were grown in Luria-Bertani medium supplemented with 30 g/mL kanamycin (Invitrogen) at 37°C to an optical density OD600 nm of 0.5, followed by induction of the fusion-protein expression with isopropyl *β*-D-thiogalactopyranoside (Invitrogen). IPTG was added at a final concentration of 1 mM and the culture was grown for additional 3 h and centrifuged (4000 ×g, 20 min, 4°C) (Hitachi Himac CR21-GIII). The pellets were resuspended in 10 mL of 0.05 M Tris/HCl, pH 8.0 buffer, and 0.15 M NaCl. Bacterial cells disruption by sonication (Unique, Campinas, Brazil) were lysed in 0.5% Triton X-114 (Sigma-Aldrich) (twelve cycles of ultrasound, 30 s, 40 W) and centrifuged (4000 ×g, 20 min, 4°C). LPS was removed by addition of chloroform (3 : 1, v/v) at 4°C after centrifugation. The protein was further purified by Ni–NTA affinity chromatography and eluted with an imidazole gradient (100–250 mM) in the same sonication buffer. The imidazole was removed by dialyzed against 0.05 M Tris/HCl, pH 8.0, and 0.15 M NaCl buffer. The His-tag was removed by digestion with 0.5 U thrombin (GE Healthcare) per milligram of fusion protein for 4 h at 18°C. The final step of purification involved molecular exclusion chromatography on a Superdex 75 HR 10/30 column (ÄKTA Purifier, GE Healthcare) in 0.05 M Tris/HCl, pH 8.0, and 0.15 M NaCl to separate the rBbKI protein from His-tag; the homogeneity of the preparation was analyzed by SDS-PAGE and on *μ*-Bondapak C18 reverse phase column (Figure 1).

### 2.3. Elastase-Induced Emphysema Mice Model

**Figure 2:**
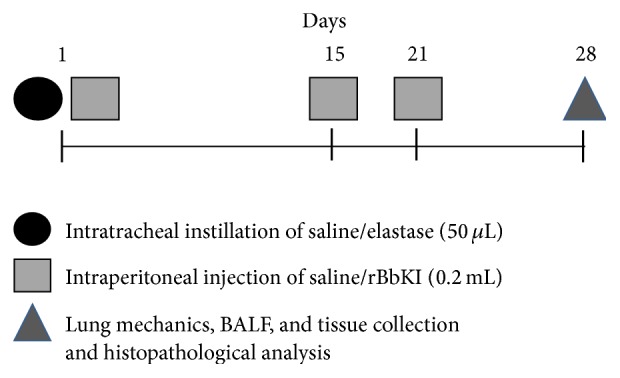
Timeline of the experimental protocol.

Seven-week-old C57Bl/6 mice were anesthetized via an intramuscular injection of ketamine (400 mg/kg) and xylazine (55 mg/kg). Each animal received an intratracheal instillation of 0.667 UI porcine pancreatic elastase (Elastase Type I/E-1250, Sigma Aldrich) diluted in 50 *µ*L of sterile saline or 50 *µ*L of sterile saline alone (control group) (Figure 2).

### 2.4. BbKI Treatment

After elastase instillation, the animals received an intraperitoneal injection of rBbKI (2 mg/kg) on days 1, 15, and 21. All doses were administered two hours after porcine pancreatic elastase instillation (Figure 2). This dose was determined in the previous studies of Brito et al. [18] which showed that this is the dose required to extrapolate the amount necessary for complete inhibition of circulating plasma kallikrein and no toxic effects was observed in the experimental model.

### 2.5. Experimental Design

Animals were divided into four groups:

(a) Animals that received intratracheal instillation and an intraperitoneal injection of saline (SAL, *n* = 8).
(b) Animals that received intratracheal instillation of elastase and an intraperitoneal injection of saline (ELA, *n* = 8).
(c) Animals that received intratracheal instillation of saline and an intraperitoneal injection of rBbKI (SAL-rBbKI, *n* = 8).
(d) Animals that received intratracheal instillation of elastase and an intraperitoneal injection of rBbKI (ELA-rBbKI, *n* = 8).

### 2.6. Mechanical Evaluation

Twenty-eight days after elastase or saline instillation, mice were anesthetized using pentobarbital sodium (50 mg/kg ip) and tracheostomized, and lung mechanics were measured using a computer controlled small animal ventilator (FlexiVent, Scireq, Montreal, Canada).

When the animal was motionless, data recording began. We used a signal that produced oscillations at different frequencies with the airflow material (0.25 to 19.625) for 16 seconds, maintaining a closed expiratory valve. Pressure values were obtained and the impedance of the airway (pressure/flow) was calculated as a function of the different frequencies.

Using a pop-up signal of 75% in 16 seconds, 3 blocks of 8 seconds were used to calculate the parameters of the mechanical oscillation (i), according to the following equation: *Z*(*f*) = *R*_aw_ + *i*(2*πf*)*l*_aw_ + [*G*_tis_ − *iH*_tis_]/(2*πf*)^*α*^.

In this model, *Z*(*f*) is the impedance of air as a function of frequency, *i* is the imaginary unit (−1.5), *f* is frequency, *l*_aw_ is the inertance of the airways, and *α* = (2/*π*)*∗*arctan⁡(*H*_tis_/*G*_tis_). The parameters experimentally obtained were airway resistance greater (*R*_aw_), lower airway resistance or tissue (*G*_tis_), and lung tissue elastance (*H*_tis_).

Animals were ventilated at 60 breaths/min with a tidal volume of 20 mL·kg^−1^. After these procedures, mice were exsanguinated and a bronchoalveolar lavage fluid analysis was performed. Afterward, the lungs were removed and fixed at a constant pressure, 20 cmH_2_O, for 24 hours in 10% formaldehyde.

### 2.7. Bronchoalveolar Lavage Fluid Analysis

Bronchoalveolar lavage fluid (BALF) analysis was performed by injecting 0.5 mL of saline three times through a tracheal cannula. Total cell analysis was determined using a* Neubauer* chamber. Cell differentiation was performed using a cytospin preparation stained with Diff-Quick Reagent (Biochemical Sciences Inc., Swedesboro, NJ). A cell differential count was performed by evaluating >250 cells using an optical microscope, using the morphologic criteria [21].

### 2.8. Morphometric Studies

Histological sections were stained with* hematoxylin-eosin* and were evaluated by researchers blinded to the protocol design. The quantification of mean linear intercept (Lm) was performed using an optical microscope with an integrated eyepiece containing a known area (10^4^ *µ*m^2^ at a magnification of 1000x) of 50 lines and 100 points. We analyzed 20 randomly selected fields for each animal.

To analyze elastic and collagen fibers, lung slices were stained with picrosirius red, a specific method for collagen detection, using the Weigert's Resorcin-Fuchsin method for elastic fibers. We measured the total area of alveolar walls and the area of collagen or elastic fibers in 15 to 20 microscopic fields per lung using a 400x magnification and the Image-ProPlus 4.5* Image Analysis System* (Media Cybernetics, Silver Spring, USA). The collagen or elastic content (%) was expressed as the relationship between the quantity of collagen or elastic fibers in a specific frame and the total area of that frame (volume fraction).

Additional slices were also prepared for immunohistochemical staining to evaluate the expression of cellular MMP-9, MMP-12, TIMP-1, MUC-5, iNOS, and eNOS.

Morphometric analysis was performed using an optical microscope with a lattice of lines and points (50 lines and 100 points) using the point counting technique. We analyzed 20 fields of lung parenchyma. The results were expressed as the % of positive area (volume fraction) [22, 23].

For airway evaluation, the reticle was coupled adjacent to the wall of the airway from the base of the epithelium. Three to five airways were randomly selected for each animal and approximately three fields per airway were evaluated. This technique was used to count neutrophils, cells positive for TNF-*α*, MMP-9, MMP-12, TIMP-1, MUC-5, eNOS, and iNOS, and the volume fraction of PGF2*α*, collagen, and elastic fibers [24].

### 2.9. Mean Linear Intercept (Lm)

The alveolar diameter analysis was performed using a microscope with an appropriate reticulum to calculate the mean alveolar diameter. The initial area of alveolar walls was determined, excluding vessels and airways. A lattice with 50 straight (100 points) lines was placed over the area of the alveolar walls and the intersections of the points in the alveolar wall were counted. Thus, the mean alveolar diameter was calculated according to the ratio of the lung parenchyma area to the number of intersections between the lines and the alveolar walls [21].

### 2.10. Immunohistochemistry

For immunohistochemistry experiments, lung sections were deparaffinized and rehydrated as follows: Step 1:* recovery of antigen*: sections were treated with Proteinase K for 20 minutes (37°C), followed by 20 minutes at room temperature. The slides were then washed in PBS. Step 2:* blocking* and* incubation with primary antibody*: blocking of endogenous peroxidases was performed by incubation with 3% hydrogen peroxide (H_2_O_2_), 100 V (3 × 10 minutes). This was followed by incubation with primary antibodies which were applied to sections of experimental and control tissue (positive and negative) and the slides were incubated overnight. Step 3:* incubation with secondary antibody and peroxidase complex*: the slides were washed in PBS and incubated with the secondary antibody using an ABCKit by Vectastain (Vector Elite-PK-6105 (anti-goat)/PK-6101 (anti-rabbit)). Step 4:* visualization*: the slides were washed in PBS and proteins were visualized using 3,3′-diaminobenzidine chromogen (DAB) (Sigma Chemical Co., St. Louis, Missouri, USA). Step 5:* counter-staining and mounting of slides*: the slides were washed thoroughly in tap water and counter-stained with* Harris Hematoxylin* (Merck, Darmstadt, Germany). The slides were then washed in water, dehydrated, cleared, and mounted using Entellan microscopy resin (Merck).

The following primary antibodies were used: anti-mouse macrophage (MAC-2) (Cedarlane Lab, Ontario, Canada; 1 : 60,000); anti-mouse neutrophils (AbD Serotec, Kidlington, UK; 1 : 400); anti-mouse matrix metalloproteinase (MMP) 9 (Santa Cruz Biotechnology, CA; 1 : 500); anti-mouse MMP-12 (Santa Cruz Biotechnology, CA; 1 : 100); anti-mouse inducible nitric oxide synthase (iNOS) (LabVision, NeoMarkers, California; 1 : 500); anti-endothelial nitric oxide synthase (eNOS) (LabVision, NeoMarkers, CA; 1 : 100); anti-tissue inhibitor of metalloproteinases (TIMP) 1 (LabVision, NeoMarkers, CA; 1 : 400); anti-tumor necrosis factor (TNF) *α* (Santa Cruz Biotechnology, California, U.S.; 1 : 100); anti-8-iso-PGF2*α* (Oxford Biomedical Research, Oxford, United Kingdom; 1 : 10.000), and anti-mucin (MUC) 5 (LabVision, NeoMarkers, CA; 1 : 400).

### 2.11. Data Analysis

Values are expressed as mean ± standard error (SEM). Statistical analysis was performed using* SigmaStat* software (SPSS Inc., Chicago, IL, USA). Data were evaluated using* One-Way Analysis of Variance* and multiple comparisons were performed using the* Holm-Sidak* method. A *P* value < 0.05 was considered statistically significant.

## 3. Results

### 3.1. Lung Mechanics

**Figure 3:**
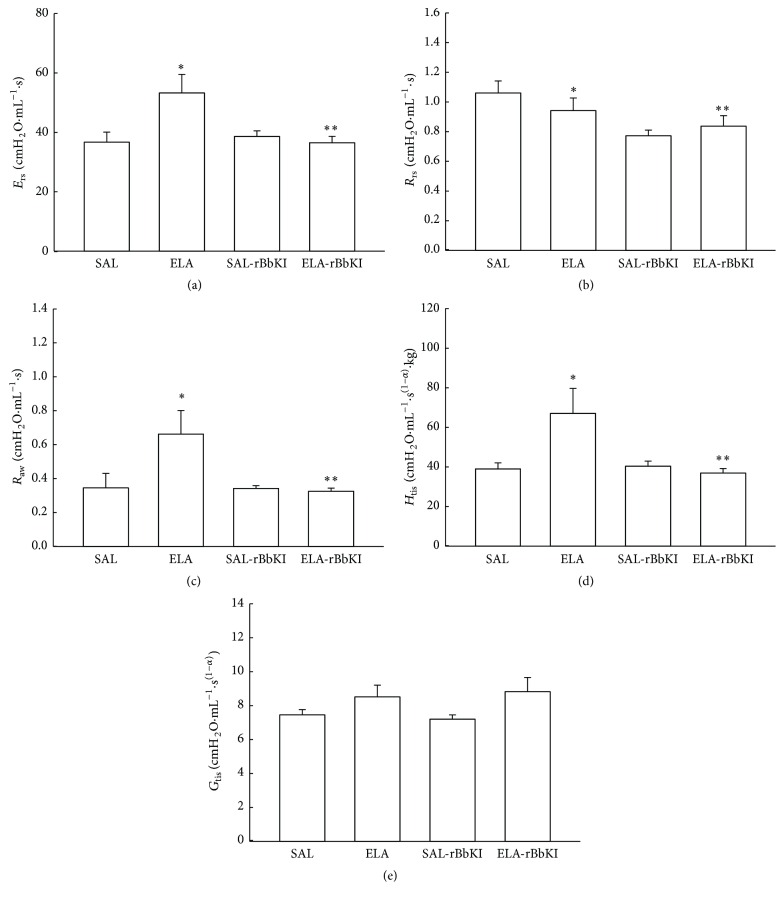
Mechanical evaluation: respiratory system elastance (a), respiratory system resistance (b), airway resistance (c), lung tissue elastance (d), and lung tissue damping (e) (^*∗*^ *P* < 0.05 compared with SAL and SAL-rBbKI groups; ^*∗∗*^ *P* < 0.05 compared with ELA-rBbKI group).

Figure 3(a) shows the values of respiratory system elastance (*E*_rs_) in all experimental groups. There was a significant increase of *E*_rs_ in ELA groups when compared with controls (*P* < 0.05, SAL: 36.99 ± 7.64 cmH_2_O·mL^−1^; ELA: 53.58 ± 15.36 cmH_2_O·mL^−1^·s; SAL-rBbKI: 38.96 ± 4.58 cmH_2_O·mL^−1^; ELA-rBbKI: 36.83 ± 5.7 cmH_2_O·mL^−1^). Additionally, there was a significant difference between ELA and ELA-rBbKI animals (*P* < 0.05).

Figure 3(b) shows the values of respiratory system resistance (*R*_rs_) in all experimental groups. There was a significant decrease of *R*_rs_ in rBbKI groups compared with the SAL group (*P* < 0.05, SAL: 1.06 ± 0.19 cmH_2_O/mL^−1^·s; ELA: 0.94 ± 0.24 cmH_2_O/mL^−1^·s; SAL-rBbKI: 0.77 ± 0.09 cmH_2_O/mL^−1^·s; ELA-rBbKI: 0.84 ± 0.18 cmH_2_O/mL^−1^·s). Animals that received elastase and were treated with rBbKI demonstrated a decrease in *R*_rs_ compared with elastase-induced mice (*P* < 0.05).

Figure 3(c) shows the values of airway resistance (*R*_aw_) in all experimental groups. There was a significant increase of *R*_aw_ in ELA groups when compared with controls (*P* < 0.05, SAL: 0.35 ± 0.22 cmH_2_O/mL/s; ELA: 0.66 ± 0.36 cmH_2_O/mL/s; SAL-rBbKI: 0.34 ± 0.04 cmH_2_O/mL/s; ELA-rBbKI: 0.33 ± 0.04 cmH_2_O/mL/s). There was a significant difference between the ELA and ELA-rBbKI groups (*P* < 0.05).

Figure 3(d) shows the values of lung tissue elastance (*H*_tis_) in all animals (SAL: 39.50 ± 6.89 cmH_2_O/mL/s^(1-*α*)^; ELA: 67.59 ± 28.18 cmH_2_O/mL/s^(1-*α*)^; SAL-rBbKI: 40.85 ± 6.19; ELA-rBbKI: 37.36 ± 6.2 cmH_2_O/mL/s^(1-*α*)^). Animals that received elastase and were treated with rBbKI had decreased *H*_tis_ compared with elastase-induced mice (*P* < 0.05).

Figure 3(e) shows the values of lung tissue damping (*G*_tis_) in all experimental groups. There was no difference between both groups (SAL: 7.50 ± 0.76 cmH_2_O/mL/s^(1-*α*)^; ELA: 8.56 ± 1.69 cmH_2_O/mL/s^(1-*α*)^; SAL-rBbKI: 7.25 ± 0.62 cmH_2_O/mL/s^(1-*α*)^; ELA-rBbKI: 8.86 ± 2.39 cmH_2_O/mL/s^(1-*α*)^).

### 3.2. Bronchoalveolar Lavage Fluid

The total and differential inflammatory cell counts are shown in Table 1. The ELA group had an increase in total inflammatory cells, macrophages, neutrophils, lymphocytes, and eosinophils compared with the SAL and SAL-rBbKI groups (*P* < 0.05). Both cell counts were significantly reduced in the ELA-rBbKI group compared with the ELA group (*P* < 0.05). There were no significant differences between the SAL, SAL-rBbKI, and ELA-rBbKI groups.

### 3.3. Mean Linear Intercept (Lm)

**Figure 4:**
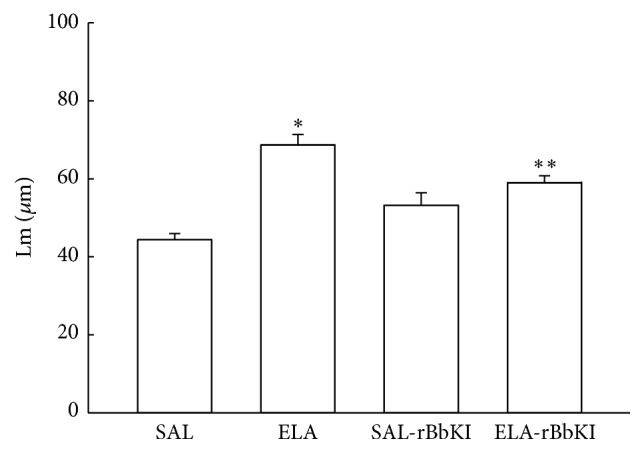
Mean linear intercept (^*∗*^ *P* < 0.05 compared with SAL and SAL-rBbKI groups; ^*∗∗*^ *P* < 0.05 compared with ELA-rBbKI group).

Figure 4 shows the Lm values in all experimental groups. There was a significant increase of Lm in the ELA group compared with SAL and SAL-rBbKI groups (*P* < 0.05). In the ELA-BbKI group we observed a reduction of Lm compared with the ELA group (*P* < 0.05). There were no significant differences between the SAL, SAL-rBbKI, and ELA-rBbKI groups.

### 3.4. Lung Inflammation

The absolute values of inflammatory markers in all experimental groups are shown in Table 2 for alveolar walls and Table 3 for airways.

In the alveolar and airway walls, there was an increase in neutrophils-positive cells in the ELA group compared with controls (*P* < 0.05). rBbKI treatment reduced the number of neutrophils-positive cells in the alveolar and airway walls in animals that received elastase (ELA-rBbKI group) compared with the ELA group (*P* < 0.05). There were no significant differences between the SAL, SAL-rBbKI, and ELA-rBbKI groups.

In the alveolar walls, there was an increase in macrophages-positive cells in the ELA group compared with controls (*P* < 0.05). rBbKI treatment reduced the number of macrophages-positive cells in the alveolar walls in animals that received elastase (ELA-rBbKI group) compared with the ELA group (*P* < 0.05). There were no significant differences between the SAL, SAL-rBbKI, and ELA-rBbKI groups.

In the alveolar and airway walls, there was an increase in TNF-*α*-positive cells in the ELA group compared with controls (*P* < 0.05). rBbKI treatment reduced the number of TNF-*α*-positive cells in the alveolar and airway walls in animals that received elastase (ELA-rBbKI group) compared with the ELA group (*P* < 0.05), but there was an increase in the ELA-rBbKI group compared with SAL and SAL-rBbKI groups (*P* < 0.05). There were no significant differences between the SAL and SAL-rBbKI groups.

### 3.5. Extracellular Matrix Remodeling

The absolute values of remodeling markers in all experimental groups are shown in Table 2 for alveolar walls and in Table 3 for airways.

In the alveolar walls, there was an increase in the volume fraction of collagen fibers in the ELA group compared with the SAL and SAL-rBbKI groups (*P* < 0.05). In the ELA-rBbKI group, there was a reduction in the volume fraction of collagen fibers compared with ELA group (*P* < 0.05). Moreover, there was an increase in the volume fraction of elastic fibers in the ELA group compared with the SAL and SAL-rBbKI groups (*P* < 0.05). In the ELA-rBbKI group, there was a reduction in the volume fraction of elastic fibers compared with ELA group (*P* < 0.05). When evaluating airway remodeling we obtained similar results; there was an increase in the volume fraction of collagen and elastic fibers in the ELA group compared with the SAL and SAL-rBbKI groups (*P* < 0.05). rBbKI treatment in the ELA-rBbKI group reduced the volume fraction of collagen and elastic fibers compared with the ELA group (*P* < 0.05). There were no significant differences between the SAL, SAL-rBbKI, and ELA-rBbKI groups.

When evaluating the MMP-9, MMP-12, and TIMP-1 positive cells in the alveolar walls, we observed an increase in MMP-9, MMP-12, and TIMP-1-positive cells in the ELA group compared with the SAL, SAL-rBbKI, and ELA-rBbKI groups (*P* < 0.05). rBbKI treatment in the ELA-rBbKI group reduced the number of MMP-9, MMP-12, and TIMP-1-positive cells compared with the ELA group (*P* < 0.05). There were no significant differences between the SAL, SAL-rBbKI, and ELA-rBbKI groups.

We obtained similar results when evaluating the airways walls. There was an increase in MMP-9, MMP-12, and TIMP-1-positive cells in the ELA group compared with the SAL and SAL-rBbKI groups (*P* < 0.05). rBbKI treatment in the ELA-rBbKI group reduced the number of MMP-9, MMP-12, and TIMP-1-positive cells compared with the ELA group (*P* < 0.05). There were no significant differences between the SAL, SAL-rBbKI, and ELA-rBbKI groups.

### 3.6. Oxidative Stress Response

The absolute values of oxidative stress markers for all experimental groups are shown in Table 2 for alveolar walls and Table 3 for the airways walls.

In the alveolar walls, there was an increase in iNOS and eNOS-positive cells in the ELA group compared with the SAL and SAL-rBbKI groups (*P* < 0.001). In the ELA-rBbKI group we observed a reduction in the number of iNOS and eNOS-positive cells compared with the ELA group (*P* < 0.05). ELA-rBbKI group increase the number of iNOS and eNOS-positive cells compared with the SAL and SAL-rBbKI groups (*P* < 0.05). There were no significant differences between the SAL and SAL-rBbKI groups.

We observed similar results in the airway walls; there was an increase in iNOS and eNOS-positive cells in the ELA group compared with the SAL and SAL-BbKI groups (*P* < 0.05). In the ELA-rBbKI group, there were a reduced number of iNOS and eNOS-positive cells compared with the ELA group (*P* < 0.001). ELA-rBbKI group increase the number of iNOS and eNOS-positive cells compared with the SAL and SAL-rBbKI groups (*P* < 0.05). There were no significant differences between the SAL and SAL-rBbKI groups.

The evaluation of the volume fraction of 8-iso-PGF2*α* in alveolar and airways walls determined that there was an increase of the volume fraction of 8-iso-PGF2*α* in the ELA group compared with the SAL and SAL-rBbKI groups (*P* < 0.05). In the ELA-rBbKI group we observed a reduction of the volume fraction of 8-iso-PGF2*α* compared with the ELA group (*P* < 0.05). ELA-rBbKI group increase the number of the volume fraction of 8-iso-PGF2*α* compared with the SAL and SAL-rBbKI groups (*P* < 0.05). There were no significant differences between the SAL and SAL-rBbKI groups.

### 3.7. Exhaled Nitric Oxide (ENO)

**Figure 5:**
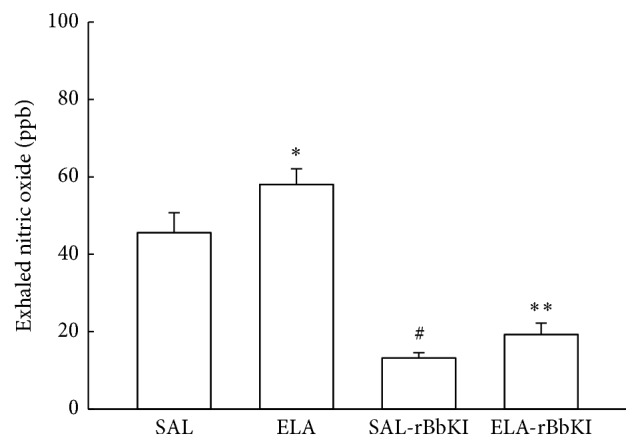
Exhaled nitric oxide (^*∗*^ *P* < 0.05 compared with SAL and SAL-rBbKI groups; ^*∗∗*^ *P* < 0.05 compared with ELA-rBbKI group; ^#^ *P* < 0.05 compared with SAL group).

Figure 5 shows the values of exhaled nitric oxide (ENO) in all experimental groups. There was a significant increase of ENO in the ELA group compared with SAL and SAL-rBbKI groups (*P* < 0.05). rBbKI treatment reduced ENO in the ELA-rBbKI group compared with ELA group (*P* < 0.05). There was a decrease of ENO in the SAL-rBbKI group when compared with the SAL group (*P* < 0.05).

### 3.8. Mucin Cells in Airway Walls

The number of positive MUC-5 cells was evaluated only in the airways and the absolute values are shown in Table 2.

There was an increase in MUC-5-positive cells in the airways in the ELA group compared with the SAL and SAL-rBbKI groups (*P* < 0.5). In the ELA-rBbKI group we observed a reduction in the number of MUC-5 positive cells compared with ELA group (*P* < 0.05). ELA-rBbKI group increase the number of MUC-5-positive cells in the airways compared with the SAL and SAL-rBbKI groups (*P* < 0.05). There were no significant differences between the SAL and SAL-rBbKI groups.

### 3.9. Qualitative Analysis

**Figure 6:**
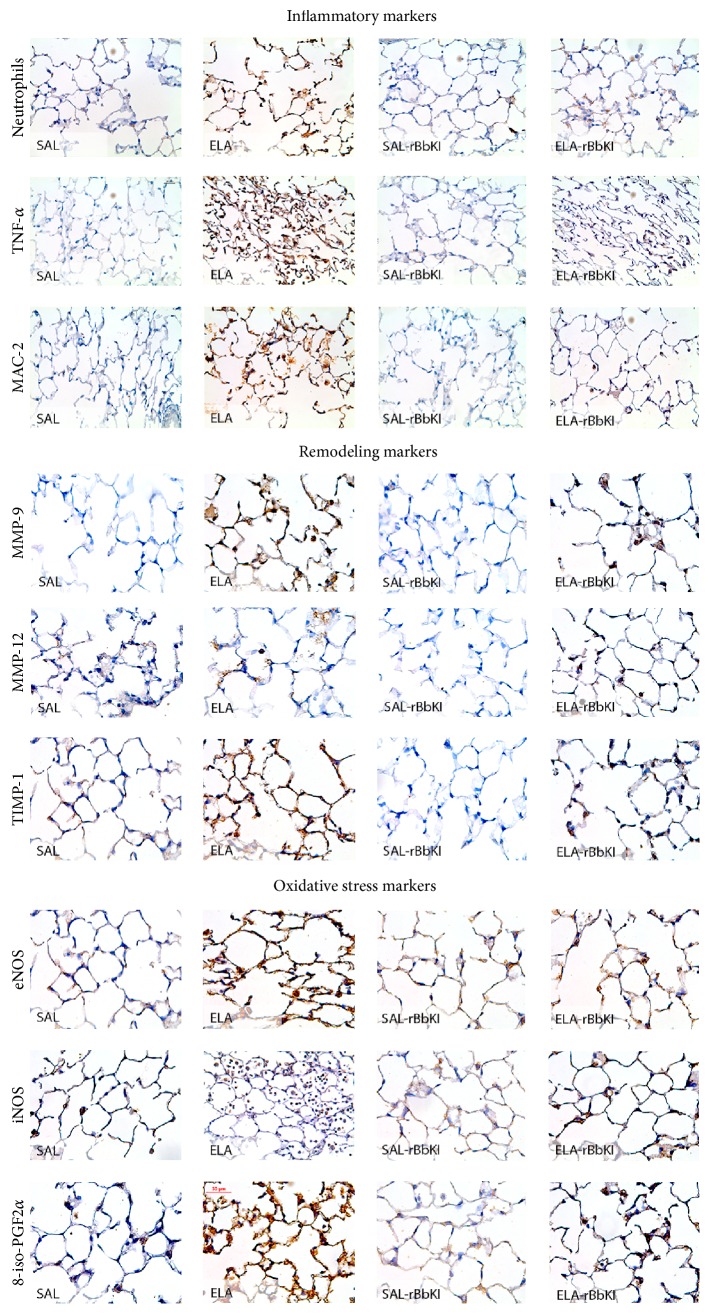
Inflammatory cells, remodeling, and oxidative stress markers in the lung tissue. Magnification of 400x.

Representative photomicrographs are presented in Figure 6, illustrating the inflammatory, remodeling, and oxidative stress processes in the alveolar septa. The slides were stained for neutrophils and cells expressing TNF-*α*, MMP-9, TIMP-1, iNOS, and 8-iso-PGF2*α*.

The ELA group exhibited increases in alveolar neutrophil infiltration, the numbers of TNF-*α*, MAC-2, MMP-9, MMP-12, TIMP-1, eNOS, and iNOS-positive cells and the volume fraction of the 8-iso-PGF2 *α*, suggesting the presence of lung inflammation, remodeling, and oxidative stress processes. rBbKI treatment reduced the above changes in alveolar walls, which can be observed in the representative photomicrographs.

## 4. Discussion

We showed that rBbKI is able to attenuate the responses of pulmonary mechanics, which has been associated with attenuation of inflammatory cells, extracellular cellular matrix (ECM) remodeling, and TNF-*α*-positive cells. There was a significant reduction in the activation of the oxidative stress pathway because we observed significant attenuation of the number of iNOS and eNOS-positive cells and of the volume fraction of 8-iso-PGF2*α* in the alveolar and airway wall in an animal model of lung induced intratracheal elastase injury.

This model of chronic obstructive pulmonary disease had been already studied in mice [21, 25]. In addition, these previous studies showed the alveolar enlarge of lung tissue, inflammation, and remodeling in mice treated with porcine pancreatic elastase, constituting an interesting model for the study of possible therapeutic strategies for emphysema [21, 26].

We found an effective decrease in alveolar enlargement in the ELA-rBbKI group, after the porcine pancreatic elastase instillation. Since Lm is a measurement of the average space between opposing alveolar walls [27] and the emphysema is characterized by alveolar wall destruction [1], the Lm increase in ELA and ELA-rBbKI groups suggests emphysema development in this experimental model and that the rBbKI treatment decreased these parenchymal lesions.

Our results demonstrated that rBbKI treatment reduced respiratory system resistance (*R*_rs_), airway resistance (*R*_aw_), and elastance of the respiratory system (*E*_rs_).

A bronchodilator effect caused by rBbKI treatment was observed in the airways, which was demonstrated by the reduction of respiratory system resistance (*R*_rs_) in the rBbKI treatment groups (Sal-rBbKI and ELA-rBbKI groups). This observation was confirmed by the airway resistance (*R*_aw_) data, which declined in rBbKI-treated animals in the elastase and saline groups. This suggests that rBbKI should be examined in future studies conducted in asthma experimental models.

The increase in respiratory system elastance (*E*_rs_) in animals receiving intratracheal elastase is an acute effect of elastase-induced lung injury. The *E*_rs_ data are corroborated by the *H*_tis_ results, which exhibited the same response pattern. These functional parameters demonstrated the effect of rBbKI in the airway and alveolar walls. However, other authors have observed different effects on lung mechanics in elastase treated animal models.

Hantos et al. [28] examined mice that received an intratracheal instillation of elastase and demonstrated a volume increase and an elastance decrease in the lung tissue. They observed no changes in airway resistance and tissue resistance. These authors concluded that the destruction of lung tissue was not associated with pulmonary mechanical alterations.

Ito et al. [25] studied mice that received porcine pancreatic elastase by nebulization. They observed a decrease in respiratory system elastance. The authors also suggested that abnormal collagen remodeling is a major determinant of pulmonary function and mechanical forces associated with emphysema.

However, Scuri et al. [29] demonstrated that elastase results in an increased production of bradykinin, leading to increased resistance and elastance of the respiratory system. The authors demonstrated that this response was not reversed by treatment with a histamine antagonist but was effectively blocked by a bradykinin B2 receptor antagonist, confirming the involvement of the kallikrein-kinin system in the process.

In addition, another important aspect that suggests the increase in the elastance and resistance of the lung tissue is the increase in the volume fraction of collagen and elastic fibers in the ELA group. The elastic and collagen fibers seem to be the major constituents of the extracellular matrix, accounting for lung tissue viscoelastic mechanical properties, and, interestingly, elastic fibers behave more linearly than collagen fibers [25].

Chung and Adcock [30] suggested that the evaluation of bronchoalveolar lavage fluid is one of the most common and effective methods for monitoring lung inflammation.

In our study, we observed an increase in the number of inflammatory cells in the elastase treated groups. These results are likely due to the role of neutrophil elastase displacement of leukocytes to the site of inflammation. There were no significant differences in the number of macrophages in bronchoalveolar lavage fluid from animals in the SAL and SAL-rBbKI groups. There was a significant decrease in the number of neutrophils, lymphocytes, and eosinophils in the ELA-rBbKI group, indicating that rBbKI has an important anti-inflammatory effect. These results were similar to that reported by Neuhof et al. [31] in the lung edema model caused by neutrophil activation; this study observed that an inhibitor of the Kunitz family elastase BbCI decreased edema to the same extent as a reference substance used for the treatment of this disease. Suzuki et al. [32] observed that curcumin, a member of the ginger family, significantly reduced the number of macrophages, neutrophils, and total cells in the BALF of mice that received intratracheal elastase.

Plantier et al. [33] evaluated the effect of growth keratinocyte (KGF) treatment in rats given intratracheal elastase. The authors demonstrated that KGF significantly decreased the total cell number of macrophages and neutrophils in bronchoalveolar lavage fluid. The results obtained with inflammatory models using animals pretreated with a BbCI inhibitor of HLE and cathepsin G and rBbKI inhibitors of plasma kallikrein and human plasma kallikrein suggest the involvement of proteinase inhibitors in the events of capture, rolling, cell adhesion, and transmigration [15].

The numbers of neutrophils, macrophages, and TNF-*α*-positive cells were increased in the alveolar and airway walls of the ELA group. rBbKI treatment decreased the numbers of these cells in alveolar and airway walls.

Our assessment of extracellular matrix remodeling was performed by evaluating collagen and elastic fibers in addition to metalloproteinase (MMP-9 and MMP-12) and TIMP-1-positive cells in the alveolar and airway walls. rBbKI treatment reduced the volume fraction of elastic fibers with less attenuation of collagen fibers in alveolar and airway walls. The study performed by Lopes et al. [34] demonstrated that elastase-induced rats had higher elastin values compared with animals that smoked for six months. The same pattern was observed for Type I collagen, but the authors found no significant differences between experimental groups. However, the expression of Type III collagen was higher in animals with six months of smoke exposure compared with mice induced by elastase.

rBbKI decreased the number of MMP-9- and MMP-12-positive cells in the alveoli and airway walls. It has been demonstrated that cathepsins can activate pro-urokinase, which activates plasminogen to form plasmin, which in turn activates metalloproteinases, primarily MMP-2 and MMP-9. Plasmin also activates elastase, which increases metalloproteinase and cathepsin. rBbKI strongly inhibits the activity of trypsin, plasmin, and HuPk (human plasma kallikrein). The inhibition of human plasma kallikrein activity by rBbKI causes a decreased release of bradykinin by decreasing the availability of HuPK [18, 19].

The MMP-9 increase resulted in excess production of its inhibitor, TIMP-1, as an attempt to repair the tissue damage. An excess of TIMP-1 in animals may interfere with cellular traffic and tissue repair and may also contribute to an increase of extracellular matrix deposition and fibrosis by inhibiting MMP-9 [35].

We observed an increase in the number of MUC-5-positive cells in animals receiving elastase, which may suggest that it is a later development in disease progression. However, mice, unlike humans, do not have true goblet cells, and this should be taken into consideration [36]. In addition, the washing steps during slide preparation may also have contributed to the appearance of clear mucus in the airways. Our results demonstrated that rRBbKI treatment reduced the number of MUC-5-positive cells.

In the oxidative stress evaluation, we demonstrated that the intratracheal elastase-induced pulmonary damage caused an increase in exhaled nitric oxide (ENO), which has been associated with increased numbers of macrophages, neutrophils, eosinophils, and lymphocytes.

Our rBbKI treatment group (ELA-rBbKI) had an attenuated oxidative stress response, with reduced expression of eNOS and iNOS positive cells in both alveolar and airway walls. There was a reduction in the volume fraction of 8-iso-PGF2*α* in alveolar and airway walls. Considering the isoprostane response, it is known that contact between oxidizing agents and the cell membrane results in lipid peroxidation of the cell membrane, which is responsible for the formation of a number of bioactive compounds known as isoprostanes, which are similar to prostaglandins [37]. Isoprostanes possess potent biological activity and are responsible for mediating aspects of oxidative injury [38].

We previously observed that iNOS inhibition attenuated hyperresponsiveness in animals with chronic allergic inflammation [39]. These results may explain the changes in airway resistance, because iNOS participates in airway modulation. These effects appear to be related to the modulation of Rho-kinase activity by iNOS [23, 24].

Consequently, the inflammatory response causes increased proteinase production of bradykinin, increasing the activation of proinflammatory cytokines and increasing eNOS and iNOS.

The kallikrein-kinin system has been reported to be important for vascular inflammatory responses (e.g., vasodilation and vascular permeability). High expression of tissue kallikrein protein has been reported to enhance tumorigenesis, cancer cell proliferation, angiogenesis, vascular permeability, and regulation of cancer cell invasion in different cancers. rBbKI induces hyperpolarization of the endothelial cell membrane and causes an increase in intracellular calcium. It also significantly reduces proliferative activity, which is independent of rBbKI-induced hyperpolarization of the cell membrane [40].

The present study had some limitations. In the current study, an analysis of the toxicity of rBbKI was not performed, but it emphasizes that this inhibitor was used in several experimental models* in vitro* [40] and* in vivo* [18] and also these authors did not identify adverse effects of the use of this inhibitor. However, further studies are necessary to assess toxicity. Our results support the importance of rBbKI treatment in lung mechanics, lung inflammatory responses, extracellular matrix remodeling, and oxidative stress pathways. Additional studies are needed to elucidate the mechanisms responsible for these changes.

## 5. Conclusions

We conclude that rBbKI treatment attenuated lung mechanics, inflammatory recruitment, remodeling of the extracellular matrix, and oxidative stress responses in an experimental lung injury model induced by intratracheal elastase administration. While further studies are required to explain the biochemical mechanism, rBbKI is a promising therapeutic strategy for the treatment of pulmonary emphysema.

## Figures and Tables

**Table 1:** Absolute values of amount of cells in bronchoalveolar lavage fluid (BALF). The total cells, macrophages, neutrophils, lymphocytes, and eosinophils are expressed in 10^4^ cells/mL. ^*∗*^ *P* < 0.05, compared with the SAL and SAL-rBbKI groups; ^*∗∗*^ *P* < 0.05, compared with the ELA-rBbKI group.

Cells	SAL	ELA	SAL-rBbKI	ELA-rBbKI
Total cells (10^4^ cells/mL)	0.79 ± 0.34	2.59 ± 0.99^*∗*^	0.40 ± 0.09	0.67 ± 0.23^*∗∗*^
Macrophages (10^4^ cells/mL)	0.78 ± 0.35	1.34 ± 0.53^*∗*^	0.37 ± 0.66	0.45 ± 0.21^*∗∗*^
Neutrophils (10^4^ cells/mL)	0.00 ± 0.00	0.87 ± 0.80^*∗*^	0.02 ± 0.05	0.18 ± 0.16^*∗∗*^
Lymphocytes (10^4^ cells/mL)	0.00 ± 0.00	0.32 ± 1.07^*∗*^	0.00 ± 0.00	0.00 ± 0.00^*∗∗*^
Eosinophils (10^4^ cells/mL)	0.00 ± 0.00	0.049 ± 0.028^*∗*^	0.0019 ± 0.0018	0.01 ± 0.01^*∗∗*^

**Table 2:** Absolute values of the morphometric analysis for inflammatory, remodeling, and oxidative stress markers, in the alveolar walls. The neutrophils, macrophages, TNF-*α*, MMP-9, MMP-12, TIMP-1, iNOS, and eNOS are expressed in positive cells/10^4^ *µ*m^2^. The collagen fiber, elastic fiber, and 8-iso-PGF2*α* are expressed in percentage (%). ^**∗**^ *P* < 0.05, compared with the SAL and SAL-rBbKI groups; ^*∗∗*^ *P* < 0.05, compared with the ELA-rBbKI group.

	SAL	ELA	SAL-rBbKI	ELA-rBbKI
Inflammatory markers				
Neutrophils (cells/10^4^ *μ*m^2^)	0.33 ± 0.07	1.35 ± 0.14^*∗*^	0.55 ± 0.03	0.83 ± 0.78^*∗∗*^
Macrophages (cells/10^4^ *μ*m^2^)	0.36 ± 0.03	1.19 ± 0.05^*∗*^	0.68 ± 0.05	0.81 ± 0.41^*∗∗*^
TNF-*α* (cells/10^4^ *μ*m^2^)	2.86 ± 0.54	12.50 ± 1.28^*∗*^	4.85 ± 0.77	9.69 ± 0.91^*∗*/*∗∗*^

Remodeling markers				
Collagen fibers (%)	9.64 ± 0.12	11.62 ± 0.83^*∗*^	7.75 ± 0.91	10.30 ± 2.85^*∗∗*^
Elastic fibers (%)	0.29 ± 0.05	0.47 ± 0.08^*∗*^	0.32 ± 0.10	0.30 ± 0.09^*∗∗*^
MMP-9 (cells/10^4^ *μ*m^2^)	6.39 ± 0.79	17.92 ± 0.86^*∗*^	7.90 ± 0.92	10,82 ± 1.96^*∗∗*^
MMP-12 (cells/10^4^ *μ*m^2^)	9.75 ± 0.92	21.46 ± 3.65^*∗*^	7.88 ± 1.32	13.21 ± 1.51^*∗∗*^
TIMP-1 (cells/10^4^ *μ*m^2^)	4.76 ± 0.53	13.58 ± 1.14^*∗*^	5.95 ± 0.60	6.80 ± 1.08^*∗∗*^

Oxidative stress markers				
iNOS (cells/10^4^ *μ*m^2^)	4.52 ± 0.47	19.60 ± 2.75^*∗*^	6.23 ± 1.24	9.82 ± 1.13^*∗*/*∗∗*^
eNOS (cells/10^4^ *μ*m^2^)	4.13 ± 0.58	12.33 ± 1.87^*∗*^	5.85 ± 0.78	7,00 ± 1.05^*∗*/*∗∗*^
8-iso-PGF2*α* (%)	1.51 ± 0.64	17.94 ± 1.30^*∗*^	1.48 ± 0.46	7.03 ± 1.25^*∗*/*∗∗*^

**Table 3:** Absolute values of the morphometric analysis for inflammatory, remodeling, and oxidative stress markers, in the airway walls. The neutrophils, TNF-*α*, MMP-9, MMP-12, TIMP-1, iNOS, eNOS, and MUC-5 are expressed in positive cells/10^4^ *µ*m^2^. The collagen fiber, elastic fiber, and 8-iso-PGF2*α* are expressed in percentage (%). ^*∗*^ *P* < 0.05, compared with the SAL and SAL-rBbKI groups; ^*∗∗*^ *P* < 0.05, compared with the ELA-rBbKI group.

	SAL	ELA	SAL-rBbKI	ELA-rBbKI
Inflammatory markers				
Neutrophils (cells/10^4^ *μ*m^2^)	2.31 ± 0.27	4.23 ± 0.44^*∗*^	2.20 ± 0.69	2.75 ± 0.46^*∗∗*^
TNF-*α* (cells/10^4^ *μ*m^2^)	5.34 ± 0.34	15.38 ± 0.44^*∗*^	6.90 ± 1.30	12.65 ± 0.538^*∗*/*∗∗*^

Remodeling markers				
Collagen fibers (%)	5.95 ± 0.10	9.40 ± 0.82^*∗*^	7.16 ± 0.69	8.64 ± 0.78^*∗∗*^
Elastic fibers (%)	0.45 ± 0.03	1.95 ± 0.08^*∗*^	0.97 ± 0.06	1.23 ± 0.05^*∗∗*^
MMP-9 (cells/10^4^ *μ*m^2^)	7.72 ± 0.87	16.57 ± 0.79^*∗*^	6.63 ± 0.79	10.59 ± 1.04^*∗∗*^
MMP-12 (cells/10^4^ *μ*m^2^)	13.45 ± 0.48	17.81 ± 1.00^*∗*^	7.04 ± 1.07	11.77 ± 0.78^*∗∗*^
TIMP-1 (cells/10^4^ *μ*m^2^)	4.34 ± 0.82	13.15 ± 1.15^*∗*^	5.20 ± 0.79	6.72 ± 1.06^*∗∗*^

Oxidative stress markers				
iNOS (cells/10^4^ *μ*m^2^)	1.30 ± 0.61	5.62 ± 0.56^*∗*^	1.16 ± 0.91	6.99 ± 0.61^*∗*/*∗∗*^
eNOS (cells/10^4^ *μ*m^2^)	3.64 ± 0.57	12.22 ± 0.25^*∗*^	1.74 ± 0.42	6.00 ± 0.32^*∗*/*∗∗*^
8-iso-PGF2*α* (%)	3.74 ± 0.79	23.47 ± 1.70^*∗*^	3.78 ± 0.52	16.19 ± 1.07^*∗*/*∗∗*^

Mucus production				
MUC-5 (cells/10^4^ *μ*m^2^)	1.53 ± 0.26	7.66 ± 0.56^*∗*^	1.32 ± 0.26	3.82 ± 0.41^*∗*/*∗∗*^

## Abbreviations

COPD: Chronic obstructive pulmonary disease
BbKI: * Bauhinia bauhinioides Kallikrein* inhibitor
rBbKI: Recombinant form of* Bauhinia bauhinioides Kallikrein* Inhibitor
ENO: Exhaled nitric oxide
BALF: Bronchoalveolar lavage fluid
Lm: Linear median
HuPk: Human plasma kallikrein
MMP-9: Metalloproteinase 9
MMP-12: Metalloproteinase 12
TIMP-1: Tissue inhibitor of metalloproteinase 1
MUC-5: Mucin 5
iNOS: Inducible nitric oxide synthase
eNOS: Endothelial nitric oxide synthase
*E*_rs_: Respiratory system elastance
*R*_rs_: Respiratory system resistance
*R*_aw_: Airway resistance
*H*_tis_: Lung tissue elastance
*G*_tis_: Lung tissue damping.
